# Burden and trends of dietary iron deficiency in the Middle East and North Africa region, 1990–2021

**DOI:** 10.3389/fnut.2024.1517478

**Published:** 2025-01-21

**Authors:** Saeid Safiri, Fatemeh Amiri, Nahid Karamzad, Mark J. M. Sullman, Ali-Asghar Kolahi, Morteza Abdollahi

**Affiliations:** ^1^Social Determinants of Health Research Center, Department of Community Medicine, Faculty of Medicine, Tabriz University of Medical Sciences, Tabriz, Iran; ^2^Clinical Research Development Unit of Tabriz Valiasr Hospital, Tabriz University of Medical Sciences, Tabriz, Iran; ^3^Neurosciences Research Center, Aging Research Institute, Tabriz University of Medical Sciences, Tabriz, Iran; ^4^Department of Persian Medicine, School of Traditional Medicine, Tabriz University of Medical Sciences, Tabriz, Iran; ^5^Nutrition Research Center, Department of Biochemistry and Diet Therapy, School of Nutrition and Food Sciences, Tabriz University of Medical Sciences, Tabriz, Iran; ^6^Department of Life and Health Sciences, University of Nicosia, Nicosia, Cyprus; ^7^Department of Social Sciences, University of Nicosia, Nicosia, Cyprus; ^8^Social Determinants of Health Research Center, Shahid Beheshti University of Medical Sciences, Tehran, Iran

**Keywords:** iron deficiency, epidemiology, prevalence, incidence, burden, global

## Abstract

**Objective:**

To assess the burden of dietary iron deficiency in the Middle East and North Africa (MENA) region, focusing on prevalence and years lived with disability (YLD) in 2021 and their changes since 1990.

**Methods:**

Data from the Global Burden of Disease (GBD) 2021 study were utilised to report counts and age-standardised rates for prevalence and YLD related to dietary iron deficiency. All estimates were accompanied by 95% uncertainty intervals (UIs).

**Results:**

In 2021, the age-standardised prevalence of dietary iron deficiency in the MENA region was 14368.2 per 100,000, representing a 26% decline since 1990. The highest age-standardised prevalence rates were observed in Yemen (30146.5), Sudan (19296.9), and Morocco (15,303) per 100,000 population. Prevalence was notably highest among children under 5 years old and women of reproductive age, with a gradual decline in older age groups. The greatest reductions in prevalence were seen among older age groups, indicating an age-related downward trend from 1990 to 2021.

**Conclusion:**

Despite some improvements, dietary iron deficiency remains a significant public health concern in the MENA region, particularly in countries with lower socio-demographic indices (SDI). Targeted interventions are essential, especially for vulnerable groups such as children and women.

## 1 Introduction

Iron is an essential micronutrient integral to various physiological processes in the human body. It plays a key role in the synthesis of hemoglobin, myoglobin, cytochrome enzymes, and several other vital proteins and enzymes. In the United States, the average daily iron intake is 15 mg for adult males and 11 mg for adult females. Certain groups, such as newborns, children, adolescents, and pregnant women, have higher dietary iron requirements, making them more susceptible to iron deficiency. This deficiency may result from insufficient iron intake or impaired absorption in the gastrointestinal tract. Iron deficiency is the most common micronutrient deficiency, affecting about 2 billion people worldwide (1). This nutritional deficiency accounts for majority of the anaemia burden, a condition which affects a high percentage of the population worldwide, posing high levels of health risks, mortality and morbidity (2). The consequences of iron deficiency are beyond anaemia, including long- and short-term effects on health, development, and productivity. It affects growth in children, cognitive abilities and performance in school age children, and working efficiency in adults, leading to global economic losses (3). Iron deficiency is particularly more harmful in pregnant women, as the risk of adverse birth outcomes is increased, including premature delivery and low birth weight (4). The intensity of this problem makes iron deficiency an important public health concern, especially in low- and middle-income countries (LMICs).

According to the Global Burden of Disease (GBD) Study 2021, iron deficiency and its consequences, particularly anaemia, exhibit a region-specific burden that varies significantly across different locations, underscoring the need for more regional assessments (2). Globally, iron deficiency has been linked to a range of social and economic challenges, including poverty, level of education, and access to medical care (5). The Middle East and North Africa (MENA) region, home to over 400 million people, has long suffered from widespread micronutrient malnutrition, particularly iron deficiency (6). This region is characterised by political and economic diversity, with both undernutrition and overnutrition present. Many MENA countries are experiencing changing nutritional patterns, known as the nutrition transition, as diets become less healthy, more processed, higher in calories, and lower in micronutrient density (7). This shift in dietary structure, combined with the region’s unique age-group composition, has significantly contributed to the burden of iron deficiency. Certain populations, including women of reproductive age, children, and the impoverished, are particularly affected due to limited access to iron-rich foods and inadequate healthcare services (8).

Sufficient dietary iron intake is necessary for the synthesis of hemoglobin and myoglobin, which aid efficient oxygen transport and enhance proper muscle functioning. However, iron absorption from dietary sources tends to vary considerably with the type of food eaten. For instance, the heme iron which is present in meat, fish and such other food products is more easily utilised by the body than the plant-based non-heme iron source, which is less bioavailable (6). In the MENA region, where plant-based diets are predominant, the reliance on non-heme iron sources contributes to higher rates of iron deficiency (9). In addition to this, the intake of tea and coffee, which contain polyphenols that lower iron absorption rate, help make the situation worse (10). Similarly, traditional beliefs, gender disparities, and the economic status are social determinants that restrict access to iron-rich food, particularly among women, especially in rural and war-torn areas (11, 12).

Iron deficiency occurs at a higher rate among women of reproductive age, infants and children under five in the MENA region due to their increased biological requirements for iron. In the case of women, the loss of iron due to menstruation and pregnancy puts them at a high risk of iron deficiency. Globally, close to a third of women in the reproductive age group are anaemic, with iron deficiency responsible for approximately 50 % of these cases (13). Recent studies have demonstrated that maternal hemoglobin levels are significantly correlated with adverse maternal and neonatal outcomes, emphasising the importance of addressing iron deficiency during reproductive years (14). In the MENA region, where maternal anaemia rates are high, specific strategies are essential to address these risks and improve health indicators. In children, iron deficiency can impair cognitive development, resulting in long-term learning and educational problems, which ultimately affects the human capital and economic development of a nation (15).

Moreover, various sociocultural factors, including dietary habits, economic status, and the availability of enriched food items and supplements, especially in conflict-affected countries like Yemen and Syria, exacerbate the problem of iron insufficiency (4, 8). Additionally, socio-economic changes, climate changes, and the loss of livelihoods further aggravate the situation by undermining the ability of affected populations to obtain quality food. Hence, there is a need for targeted interventions that address both immediate and underlying causes of iron deficiency (16–18).

The GBD study provides comprehensive data on the burden of iron deficiency, and understanding these trends is critical for developing effective public health interventions. The issue of iron deficiency is not solely a personal health concern, it also impacts national growth and development, productivity, and health equity. For example, in MENA countries, large portions of the population are engaged in agriculture and other labour-intensive sectors, where efficiency depends on workers’ health and nutrition. Iron deficiency can result in reduced work capacity, higher absenteeism, and increased healthcare costs, which in turn affect economic development (15, 19).

This paper provides a detailed assessment of the burden of dietary iron deficiency in the MENA region, focusing on incidence, prevalence and years lived with disability (YLD) using GBD 2021 data. The aim is to underscore the significant public health challenges caused by iron deficiency and to offer evidence to support effective policy actions and nutritional programmes.

## 2 Methods

### 2.1 Overview

The latest version of the GBD project, GBD 2021, assessed the burden of 371 diseases and injuries across 21 regions, seven super-regions, and 204 countries and territories from 1990 to 2021. Detailed information on the GBD 2021 methods and the improvements made since GBD 2019 can be found in other references (20).

### 2.2 Case definition and data inputs

Dietary iron deficiency is defined in the GBD 2021 analysis as anaemia caused by insufficient dietary intake of iron. This does not include anaemia resulting from other factors like infections, blood loss, or genetic disorders affecting iron metabolism. The GBD anaemia causal attribution model categorises iron deficiency anaemia into mild, moderate, or severe stages based on haemoglobin levels, which are assessed using the haemoglobin concentration data from various global surveys (20).

The data inputs for estimating dietary iron deficiency include individual-level haemoglobin concentration data from a variety of sources such as the *WHO Vitamin and Mineral Nutrition Information System* database, population-based surveys (e.g., DHS), and published cohort studies. These data were processed to estimate the prevalence of anaemia by severity (mild, moderate, severe) across different regions, age groups, and sexes. The GBD model used inputs like haemoglobin means and standard deviations, along with covariates such as the Socio-demographic Index (SDI), to produce reliable estimates for each population group. The sources are accessible through the GBD 2021 Data Input Sources Tool.^1^ However, variations existed in the types of databases utilised across certain regions.

### 2.3 Data processing and disease model

Dietary iron deficiency was evaluated as part of the *GBD Anaemia Causal Attribution* framework. This framework consists of two key components: the estimation of the anaemia envelope and causal attribution, both of which influence iron deficiency estimates. Detailed methods for the analysis can be found in the description of “Anaemia (Impairment)” (20).

In the first stage, the anaemia envelope was determined by estimating the prevalence of mild, moderate, and severe anaemia across different GBD locations, age groups, sexes, and years. The model used mean haemoglobin (Hb) concentration and its standard deviation (SD) as key inputs. The mean Hb was directly modelled using spatio-temporal Gaussian process regression (ST-GPR), while the SD was derived from a variance optimisation process that incorporated the modelled mean Hb and prevalence of severe, moderate-to-severe, and total anaemia. For each population group, defined by location, year, age, and sex, the model’s haemoglobin distribution was adjusted to minimise error between the predicted and observed prevalence of anaemia. Next, individual-level data were used to create a set of ensemble distribution weights using the method of moments. These weights, combined with the mean and SD, produced haemoglobin distribution estimates for each population group (20).

The second step, anaemia causal attribution, generated counterfactual haemoglobin distributions based on the prevalence or incidence of anaemia causes, such as maternal haemorrhage. Meta-analysis determined the cause-specific haemoglobin shifts. The same ensemble distribution weights were used for both the anaemia envelope and sub-causes, as there was insufficient data to customise distributions for each cause. Mild, moderate, and severe anaemia levels were attributed to each cause based on the difference between observed and counterfactual distributions.

Any remaining residual anaemia cases were then distributed among five GBD causes: (1) dietary iron deficiency, (2) haemoglobinopathies and haemolytic anaemias, (3) infectious diseases, (4) neglected tropical diseases, and (5) endocrine, metabolic, blood, and immune disorders. The distinction between “dietary iron deficiency” as a GBD cause and “iron deficiency” as a GBD risk is crucial. Many anaemia-causing conditions lead to iron deficiency symptoms without being caused by insufficient iron intake, such as hookworm, malaria, gastrointestinal disorders, or vitamin A deficiency. The term “dietary iron deficiency” specifically refers to anaemia caused by insufficient dietary iron intake, as opposed to conditions caused by blood loss or iron metabolism disorders.

Estimates should be interpreted cautiously, as they may include some acute and chronic blood loss conditions where supplementation could be beneficial, but poor iron intake is not the sole cause. Our long-term objective is to integrate all anaemia causes into the GBD Anaemia Causal Attribution framework and reduce the reliance on residual categories (20).

### 2.4 Disability weight and severity distribution

The disability weights (DWs) for mild [0.004 (0.001–0.008)], moderate [0.052 (0.034–0.076)], and severe [0.149 (0.101–0.209)] anaemia were derived from the GBD 2013 European Disability Weights Measurement Study. The prevalence for each level of severity was then multiplied by its corresponding DW to calculate the YLDs. Since no deaths could be directly linked to anaemia, years of life lost (YLLs) were not estimated, making YLDs equivalent to disability-adjusted life years. Age-standardised rates, including point prevalence and YLDs, were reported per 100,000 population using the GBD standard population. To account for uncertainty, 1,000 samples were drawn at each computational step, incorporating uncertainties from input data, measurement error adjustments, and residual non-sampling errors. The uncertainty intervals (UIs) were defined as the 2.5th and 97.5th percentiles of these 1,000 draws.

### 2.5 Compilation of results

The relationship between anaemia burden, measured in YLDs, and the SDI was analysed using smoothing spline models across countries. SDI, an indicator of a nation’s development, integrates factors such as lag-adjusted income per capita, the 10-year smoothed gross domestic product per capita, educational attainment in individuals aged 15 and older, and the total fertility rate for those under 25 years of age. SDI values range from 0 (least developed) to 1 (most developed). All analyses were done using R software (version 3.5.2).

## 3 Results

### 3.1 The MENA region

In 2021, the MENA region recorded 88,916,154 prevalent cases of iron deficiency (95% UI: 85,860,972 to 92,220,594), yielding an age-standardised prevalence of 14368.2 per 100,000 individuals (95% UI: 13897.3 to 14893.4). This represents no significant change since 1990, with a decline of 26% (95% UI: −29.2% to −23.0%) (Table 1). Iron deficiency resulted in 2,241,355 YLD (95% UI: 1,501,840 to 3,227,407), equating to an age-standardised rate of 359.7 per 100,000 (95% UI: 241.8 to 517.4), also indicating no significant change since 1990 [−28% (95% UI: −31.5 to −24.5)] (Table 1).

**Table 1:** Prevalence and YLDs due to dietary iron deficiency in 2021 and percentage change of age-standardised rates during 1990–2021 (generated from data available from http://ghdx.healthdata.org/gbd-results-tool).

	Prevalence (95% UI)					YLD (95% UI)				
	Counts (1990)	ASRs (1990)	Counts (2021)	ASRs (2021)	Pcs in ASRs 1990–2021	Counts (1990)	ASRs (1990)	Counts (2021)	ASRs (2021)	Pcs in ASRs 1990–2021
North Africa and Middle East	67,719,382 (65,189,472, 70,845,871)	19417.4 (18758.6, 20143.5)	88,916,154 (85,860,972, 92,220,594)	14368.2 (13897.3, 14893.4)	−26 (−29.2, −23)	1,841,629 (1,208,726, 2,630,679)	499.6 (331.5, 711.2)	2,241,355 (1,501,840, 3,227,407)	359.7 (241.8, 517.4)	−28 (−31.5, −24.5)
Afghanistan	1,818,630 (1,661,855, 1,989,564)	18377.4 (16,980, 19,936)	4,182,706 (3,812,993, 4,571,011)	14128.9 (13051.2, 15121.8)	−23.1 (−31.1, −14.7)	55,422 (35,102, 80,406)	528.2 (341.2, 761.8)	111,700 (70,810, 164,019)	356.5 (231, 510.2)	−32.5 (−44.6, −17.8)
Algeria	5,405,567 (4,747,870, 6,277,085)	21030.8 (18649.3, 23756.4)	6,770,204 (6,057,342, 7,826,644)	15298.2 (13727.5, 17662.9)	−27.3 (−36.8, −15.5)	130,814 (82,630, 187,789)	483.4 (308.1, 689.4)	144,691 (89,274, 217,374)	323.3 (200.4, 485.7)	−33.1 (−44.9, −18.4)
Bahrain	92,220 (80,706, 106,823)	17613.7 (15607.6, 19804.9)	163,989 (141,612, 192,365)	11159.8 (9668.2, 12945.7)	−36.6 (−46.6, −22.5)	1727 (1,070, 2,639)	334 (208.1, 501.8)	2,696 (1,693, 4,180)	190.9 (122, 293.8)	−42.8 (−54.2, −30.1)
Egypt	10,885,471 (9,877,789, 12,196,326)	19251.2 (17552.7, 21332.7)	14,576,135 (13,446,669, 16,136,604)	14065.2 (12983.1, 15523.4)	−26.9 (−34.9, −17.4)	244,038 (152,924, 367,900)	411.3 (259.3, 614.9)	268,967 (169,246, 403,189)	253.4 (161, 378.9)	−38.4 (−48.4, −27.8)
Iran	9,966,093 (9,018,411, 10,939,813)	17,207 (15882.4, 18660.6)	9,584,308 (8,735,393, 10,552,132)	11432.5 (10403.3, 12468.2)	−33.6 (−40.1, −25.2)	262,507 (173,147, 387,884)	425.5 (285.7, 620.9)	231,119 (151,469, 347,184)	275.6 (182.9, 412.2)	−35.2 (−45.1, −22.1)
Iraq	3,509,178 (3,135,588, 3,950,210)	18,069 (16235.1, 20171.4)	5,330,367 (4,695,649, 5,965,128)	12942.9 (11550.2, 14369.7)	−28.4 (−37.4, −18)	92,674 (59,929, 136,855)	443.4 (285, 649.1)	120,041 (73,472, 180,931)	288.2 (176.7, 431.8)	−35 (−46.7, −21.2)
Jordan	728,685 (656,447, 826,214)	18928.8 (17,432, 21013.2)	1,826,678 (1,695,378, 1,979,105)	14886.2 (13893.4, 15992.3)	−21.4 (−30.3, −12.1)	19,711 (12,303, 29,460)	497.5 (318.3, 740)	43,386 (27,740, 65,650)	356.6 (229, 536.9)	−28.3 (−38.9, −15.5)
Kuwait	255,749 (232,496, 282,903)	13879.5 (12710.4, 15206.9)	482,744 (426,924, 560,524)	10350.8 (9316.5, 11947.1)	−25.4 (−34.9, −12.1)	4,708 (2,954, 7,058)	258.9 (162.7, 381.7)	9,594 (5,969, 14,837)	207.3 (131.3, 311.9)	−19.9 (−32.8, −2.7)
Lebanon	514,459 (472,844, 585,668)	16717.1 (15464.3, 18,709)	613,123 (559,883, 671,687)	11244.8 (10258.3, 12402.6)	−32.7 (−41.4, −24.1)	13,726 (8,836, 20,322)	439.5 (282.6, 641.8)	15,582 (10,028, 23,184)	285.6 (183.8, 419.4)	−35 (−46.2, −22.7)
Libya	696,527 (617,998, 816,398)	15705.3 (14099.2, 17,995)	851,968 (762,328, 964,327)	12773.1 (11,505, 14239.4)	−18.7 (−31, −5.2)	15,603 (9,700, 23,434)	335.6 (208.3, 494.8)	18,481 (11,542, 28,064)	283.3 (181.1, 429.8)	−15.6 (−31.2, 3.4)
Morocco	5,384,121 (4,770,927, 6,141,295)	20591.8 (18313.9, 23229.9)	5,627,604 (5,086,307, 6,328,680)	15,303 (13825.2, 17,197)	−25.7 (−35.6, −14.5)	136,680 (88,577, 199,944)	505.6 (327.5, 729.8)	127,745 (83,979, 192,242)	350.7 (231.4, 525.8)	−30.6 (−42.3, −16.8)
Oman	477,048 (419,099, 538,423)	21671.8 (19284.8, 24372.9)	571,069 (488,296, 690,375)	12519.8 (10729.6, 15393.4)	−42.2 (−52.8, −27)	7,650 (4,703, 12,551)	324.7 (201, 517.7)	8,351 (4,989, 13,101)	182.4 (109.2, 284.7)	−43.8 (−56.6, −28)
Palestine	345,888 (307,268, 399,961)	16,494 (14896.7, 18,391)	583,251 (528,441, 657,254)	11484.5 (10536.9, 12678.5)	−30.4 (−39.5, −18.7)	7,700 (4,885, 11,956)	339.5 (221.5, 512.4)	10,946 (6,720, 16,599)	214 (131.1, 321.7)	−37 (−48.6, −22.8)
Qatar	66,639 (58,710, 76,705)	14841.7 (13328.8, 16755.3)	252,352 (218,069, 300,650)	9061.9 (8112.2, 10294.5)	−38.9 (−47.4, −28.2)	1,318 (835, 1970)	295.9 (186.5, 435.4)	4,218 (2,537, 6,483)	157.9 (99, 235.9)	−46.6 (−55.8, −37.8)
Saudi Arabia	2,518,193 (2,336,590, 2,713,641)	15848.4 (14680.8, 17051.1)	3,963,169 (3,489,194, 4,627,041)	10747.9 (9692.8, 12160.4)	−32.2 (−40.5, −22.8)	68,855 (43,955, 99,452)	394.4 (252.1, 572.6)	78,540 (49,580, 117,452)	219.1 (140.9, 315.7)	−44.5 (−54.5, −32.7)
Sudan	5,112,440 (4,647,143, 5,638,137)	24539.8 (22473.1, 26970.6)	8,499,453 (7,560,679, 9,783,418)	19296.9 (17402.6, 21810.1)	−21.4 (−31, −10.4)	156,263 (98,683, 231,535)	692.7 (443, 1012.1)	218,147 (137,865, 336,682)	480.8 (308.4, 732.2)	−30.6 (−42.3, −16.6)
Syrian Arab Republic	2,716,420 (2,400,449, 3,072,516)	20955.4 (18,822, 23309.1)	2,070,604 (1,845,434, 2,369,899)	15687.1 (14033.6, 17,777)	−25.1 (−35.1, −14.8)	69,546 (43,909, 105,268)	501.9 (317.4, 745.7)	46,382 (28,820, 66,518)	350.1 (222.5, 499.5)	−30.2 (−42.4, −17)
Tunisia	1,223,498 (1,143,329, 1,325,625)	14559.1 (13621.8, 15586.9)	1,319,921 (1,196,646, 1,453,930)	11245.5 (10232.9, 12,426)	−22.8 (−31.4, −14.1)	36,190 (23,761, 52,084)	419.4 (277.7, 603)	34,403 (23,253, 49,982)	293.6 (198.7, 428.2)	−30 (−41.4, −16.2)
Turkey	11,392,720 (10,233,611, 12,943,576)	19447.6 (17590.6, 21835.1)	9,824,949 (8,790,707, 11,065,292)	12013.4 (10751.9, 13539.3)	−38.2 (−47.4, −28)	278,862 (175,570, 409,647)	472.5 (301.8, 696.7)	207,343 (134,628, 301,371)	254.3 (165.6, 367.4)	−46.2 (−55.4, −35.3)
United Arab Emirates	359,399 (317,034, 411,391)	18488.5 (16449.1, 21,081)	1,132,697 (927,291, 1,360,316)	13705.8 (11702.8, 16035.3)	−25.9 (−39, −11)	5,376 (3,377, 8,367)	277.2 (177.1, 422.6)	16,391 (9,739, 25,334)	217.9 (132.4, 329)	−21.4 (−34.4, −2.9)
Yemen	4,213,391 (3,966,827, 4,453,808)	28857.8 (27349.2, 30485.2)	10,605,932 (10,073,175, 11,136,657)	30146.5 (28766.8, 31604.7)	4.5 (−1.9, 12.7)	231,251 (159,510, 317,041)	1440.8 (988.5, 1960.9)	520,540 (361,138, 715,904)	1405.2 (980.2, 1922.8)	−2.5 (−11.7, 7.2)

### 3.2 Country-level

**Figure 1:**
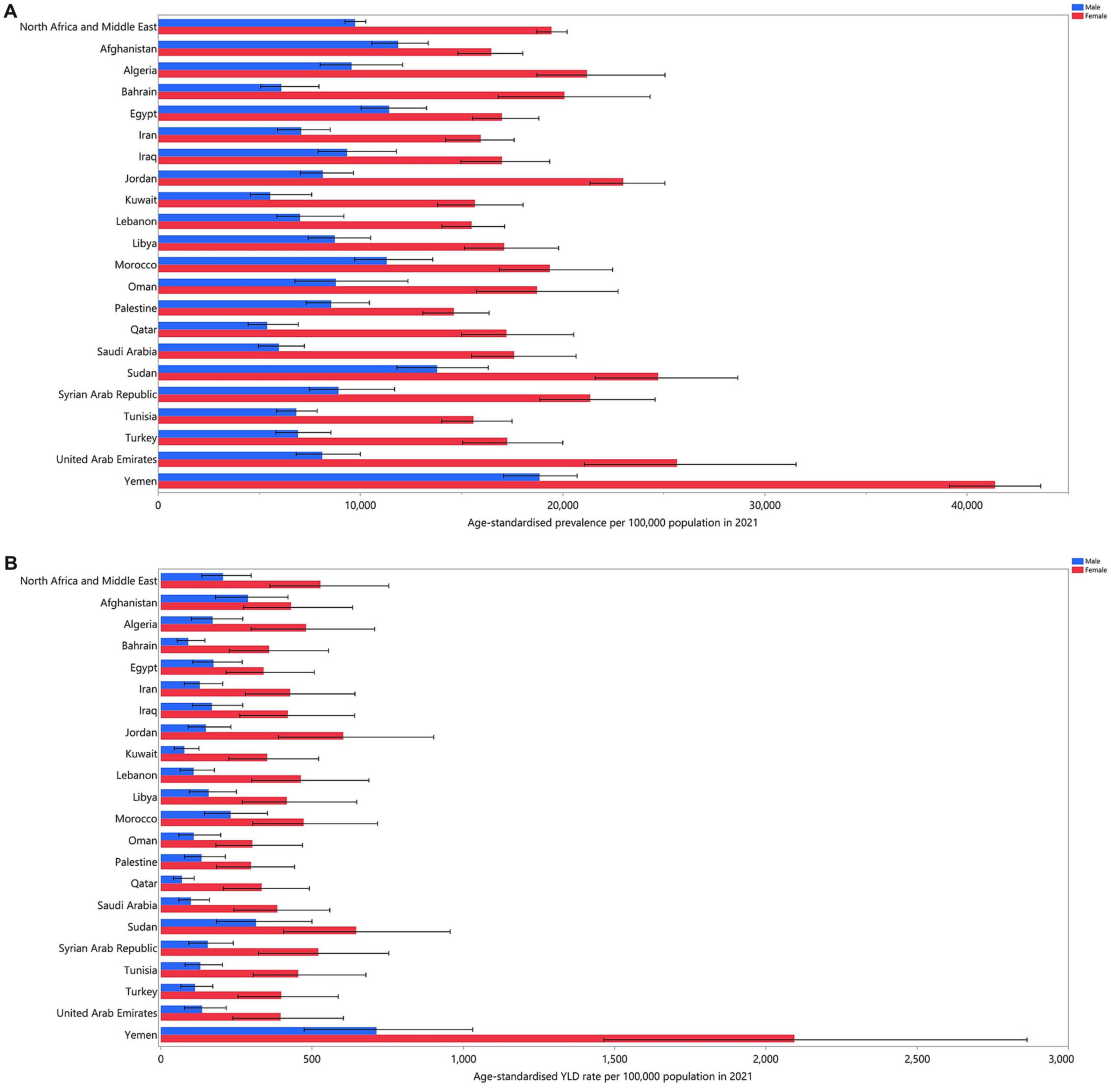
Age-standardised prevalence **(A)**, and YLDs **(B)** for dietary iron deficiency (per 100,000 populations) in the MENA region in 2021, by sex and country. YLD, years lived with disability (generated from data available from http://ghdx.healthdata.org/gbd-results-tool).

In 2021, the prevalence of iron deficiency differed between countries, with the highest number reaching 14,576,135 cases and the lowest recorded at 163,989 patients. The age-standardised prevalence ranged from a high of 30146.5 to a low of 9061.9 cases per 100,000 individuals. Yemen (30146.5; 95% UI: 28766.8 to 31604.7), Sudan (19296.9; 95% UI: 17402.6 to 21810.1), and Morocco (15303.0; 95% UI: 13825.2 to 17,197) exhibited the highest age-standardised prevalence, while Qatar (9061.9; 95% UI: 8112.2 to 10294.5), Kuwait (10350.8; 95% UI: 9316.5 to 11947.1), and Saudi Arabia (10747.9; 95% UI: 9692.8 to 12160.4) reported the lowest rates (Table 1). Furthermore, in all MENA countries, the age-standardised prevalence of dietary iron deficiency was higher in females than in males. Figure 1A reports the prevalence estimates of dietary iron deficiency by sex across countries in the MENA region.

In 2021, the age-standardised YLD rates due to iron deficiency ranged from 157.9 to 1405.2 cases per 100,000 population. The three countries with the highest rates were Yemen (1405.2; 95% UI: 980.2 to 1922.8), Sudan (1922.8; 95% UI: 308.4 to 732.2), and Jordan (356.6; 95% UI: 229 to 536.9). Conversely, Qatar (157.9; 95% UI: 99 to 235.9), Oman (182.4; 95% UI: 109.2 to 284.7), and Bahrain (190.9; 95% UI: 122 to 293.8) had the lowest rates (Table 1). Moreover, in all MENA countries, the age-standardised YLDs attributable to dietary iron deficiency were higher in females than in males. The YLD rates for dietary iron deficiency by sex in countries across the MENA region are reported in Figure 1B.

**Figure 2:**
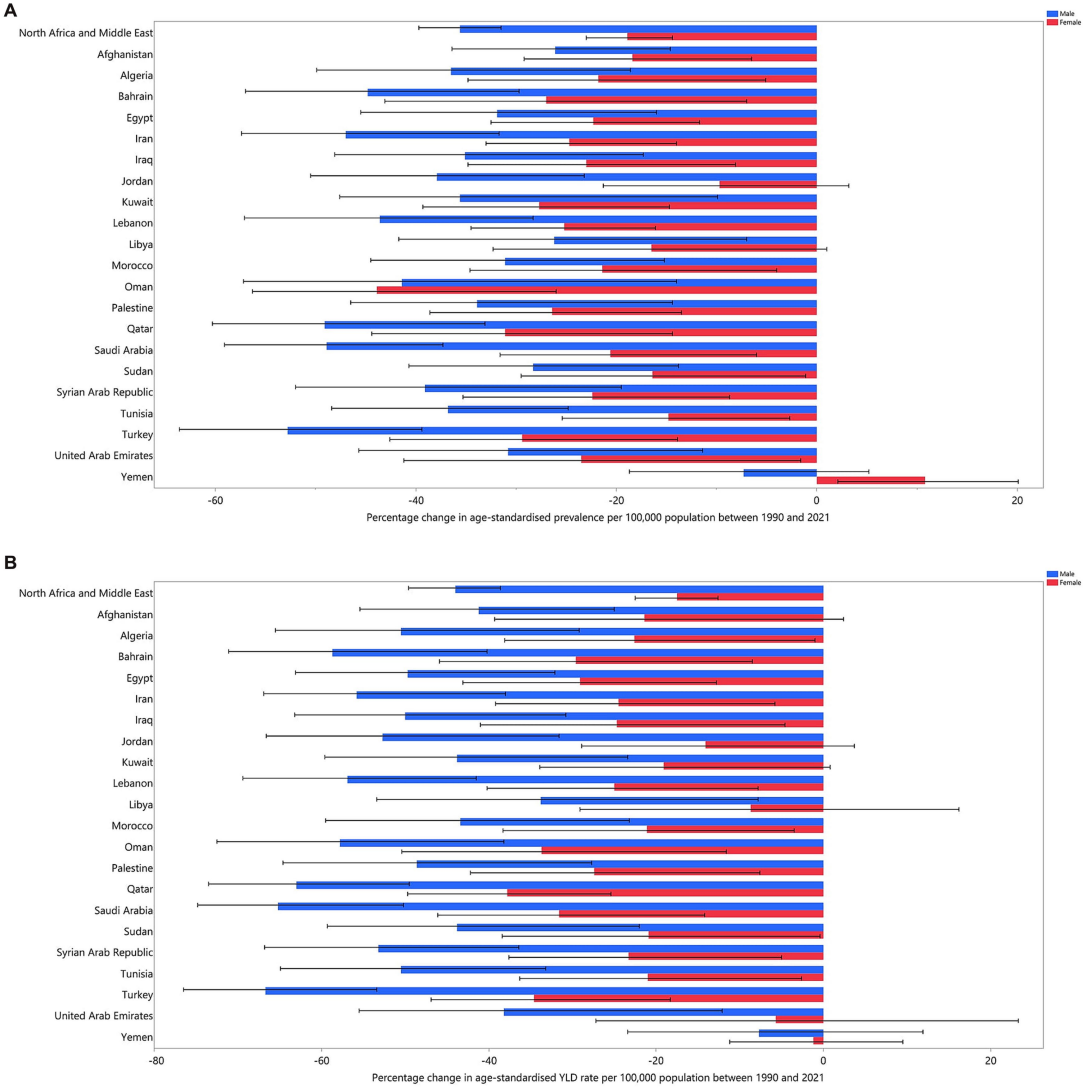
Percentage change in the age-standardised prevalence **(A)**, and YLDs **(B)** for dietary iron deficiency (per 100,000 populations) in the MENA region from 1990 to 2021, by sex and country. YLD, years lived with disability (generated from data available from http://ghdx.healthdata.org/gbd-results-tool).

In 2021, the prevalence of iron deficiency across countries in the MENA region showed significant decreases compared to 1990. The three countries with the greatest decreases in age-standardised prevalence from 1990 to 2021 were Oman, which experienced a decline of 42.2% (95% UI: −52.8% to −27%), Qatar with a decrease of 38.9% (95% UI: −47.4% to −28.2%), and Bahrain, which saw a decline of 36.6% (95% UI: −46.6% to −22.5%) (Table 1). The percentage changes in age-standardised prevalence of dietary iron deficiency from 1990 to 2021 are illustrated in Figure 2A. This indicates that, in all MENA countries except Oman, there was a larger reduction in the age-standardised prevalence of dietary iron deficiency among men compared to women between 1990 and 2021.

In 2021, the YLD rate across countries in the MENA region demonstrated substantial reductions compared to 1990. The three countries with the most significant declines in age-standardised YLD rate from 1990 to 2021 were Qatar, which experienced a reduction of 46.6% (95% UI: −55.8% to −37.8%), Turkey with a decrease of 46.2% (95% UI: −55.4% to −35.3%), and Saudi Arabia, which saw a decline of 44.5% (95% UI: −54.5% to −32.7%). Furthermore, in all MENA countries, the age-standardised YLD rate attributable to dietary iron deficiency saw a larger reduction among men compared to women between 1990 and 2021. The percentage changes in age-standardised YLD rate from 1990 to 2021 are depicted in Figure 2B.

### 3.3 Age and sex patterns

**Figure 3:**
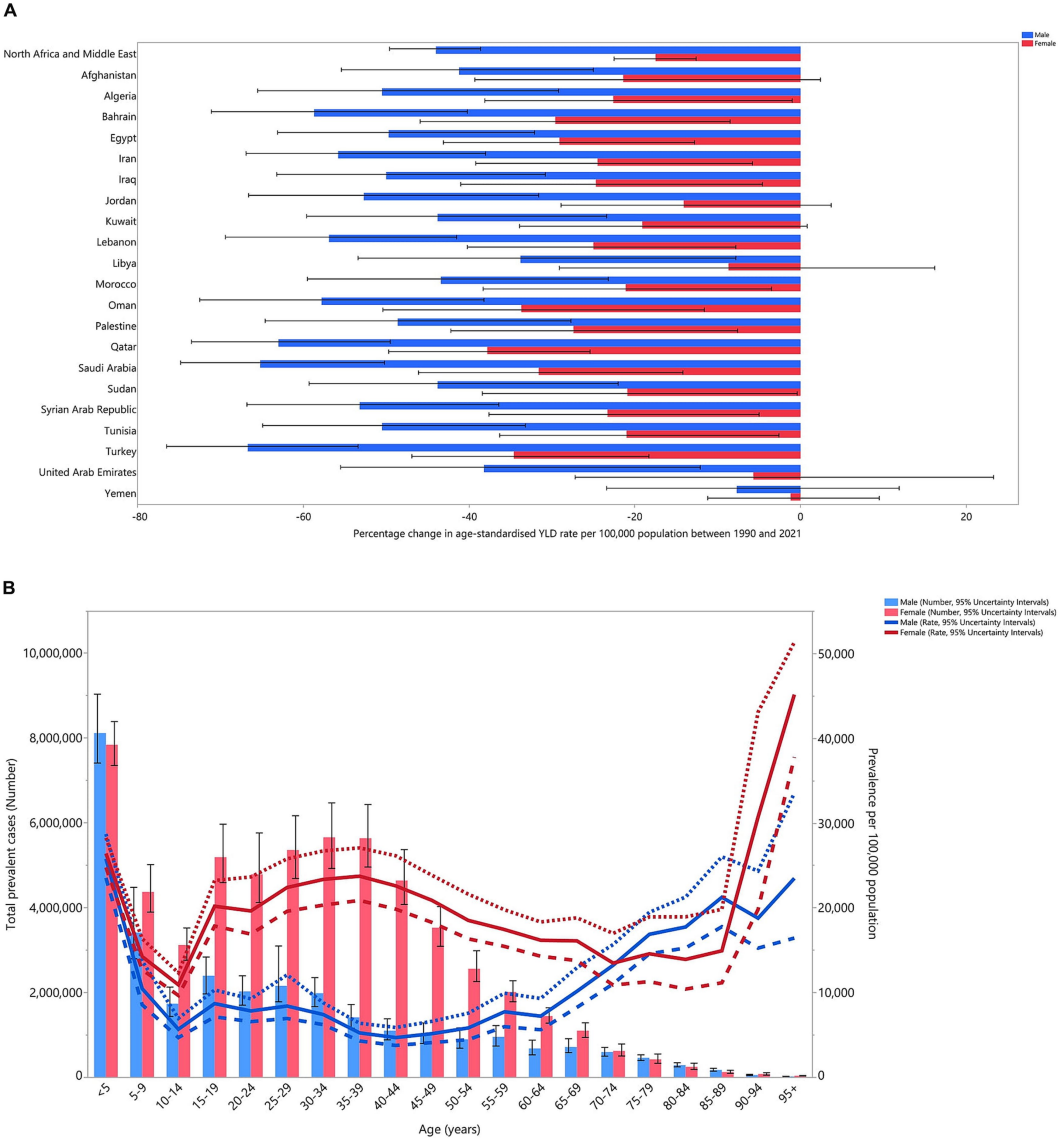
Number of prevalent cases and prevalence **(A)**, and the number of YLDs and YLD rate **(B)** for dietary iron deficiency (per 100,000 populations) in the MENA region, by age and sex in 2021; dotted and dashed lines indicate 95% upper and lower uncertainty intervals, respectively. YLD, years lived with disability (generated from data available from http://ghdx.healthdata.org/gbd-results-tool).

In 2021, the highest number of prevalent cases of iron deficiency cases in the MENA region was observed in children under 5 years of age for both sexes. Across most age groups, females had a higher prevalence than males (Figure 3A). Similarly, the highest number of YLDs were recorded in the under-5 age group for both sexes, with females showing higher YLD rates in the majority of age groups (Figure 3B).

**Figure 4:**
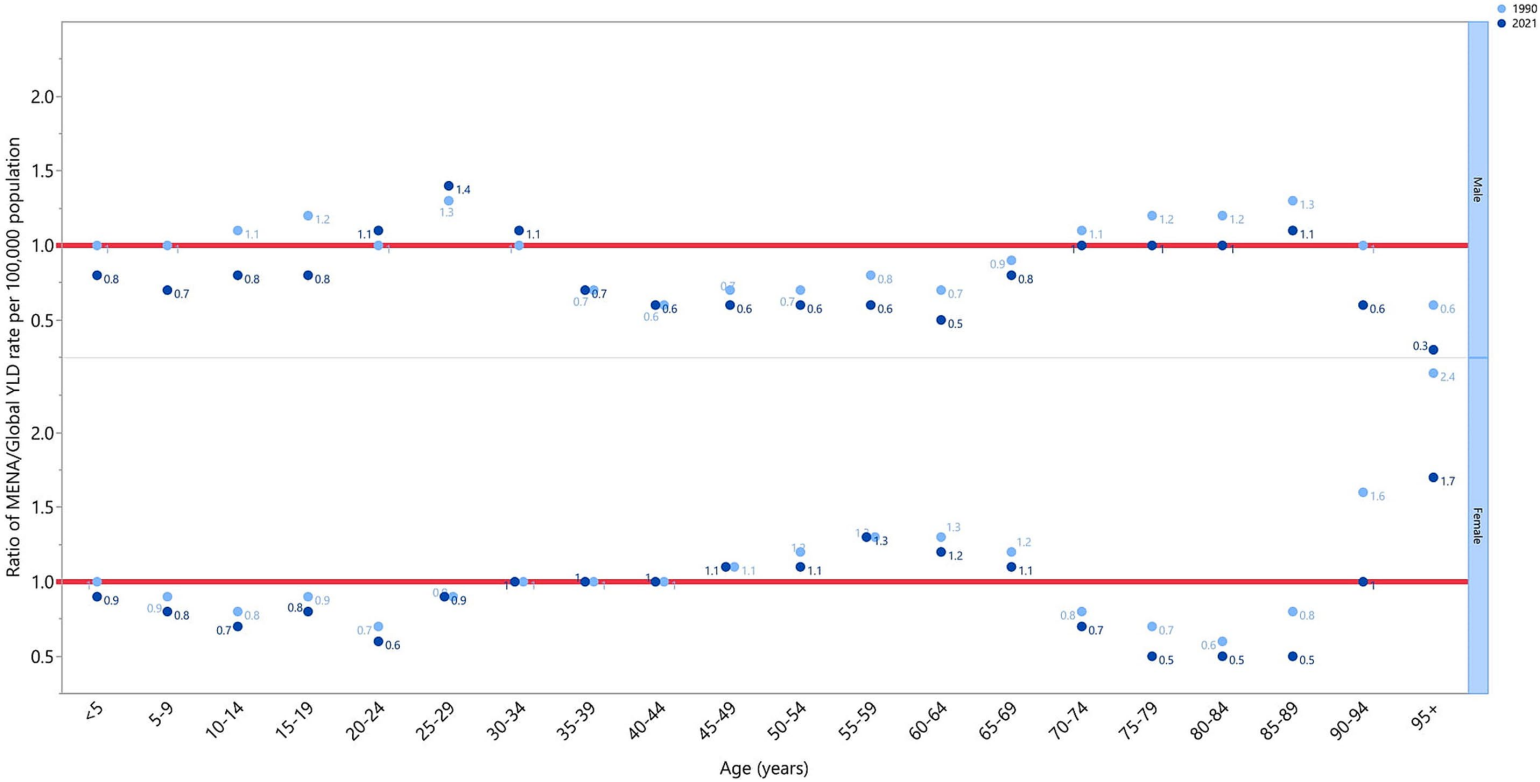
The ratio of the MENA region to global dietary iron deficiency YLD rate according to age group and sex, 1990–2021. YLD, years lived with disability (generated from data available from http://ghdx.healthdata.org/gbd-results-tool).

In 2021, males aged 20–34 and 85–89 had YLD rates above the global average (MENA/global YLD ratio >1). In females, the 45–69 and ≥95 age groups exhibited higher-than-average YLD rates in 2021. Males aged 70–84 and females aged 30–44 and 90–94 had YLD rates aligned with the global average (MENA/global YLD ratio = 1). Below-average rates (MENA/global YLD ratio <1) were found in males aged 5–19, 35–69, and ≥90, while females in the under-29 and 70–89 age groups also had below-average rates in 2021. Notably, the highest regional-to-global YLD ratios were 1.7 for females aged ≥95 and 1.4 for males aged 25–29. Comparing YLD rates from 1990 to 2021, males aged <19 and >45 and females aged <24, 50–54, and >60 had higher rates in 1990 (Figure 4).

### 3.4 Correlation with the Socio-demographic Index

**Figure 5:**
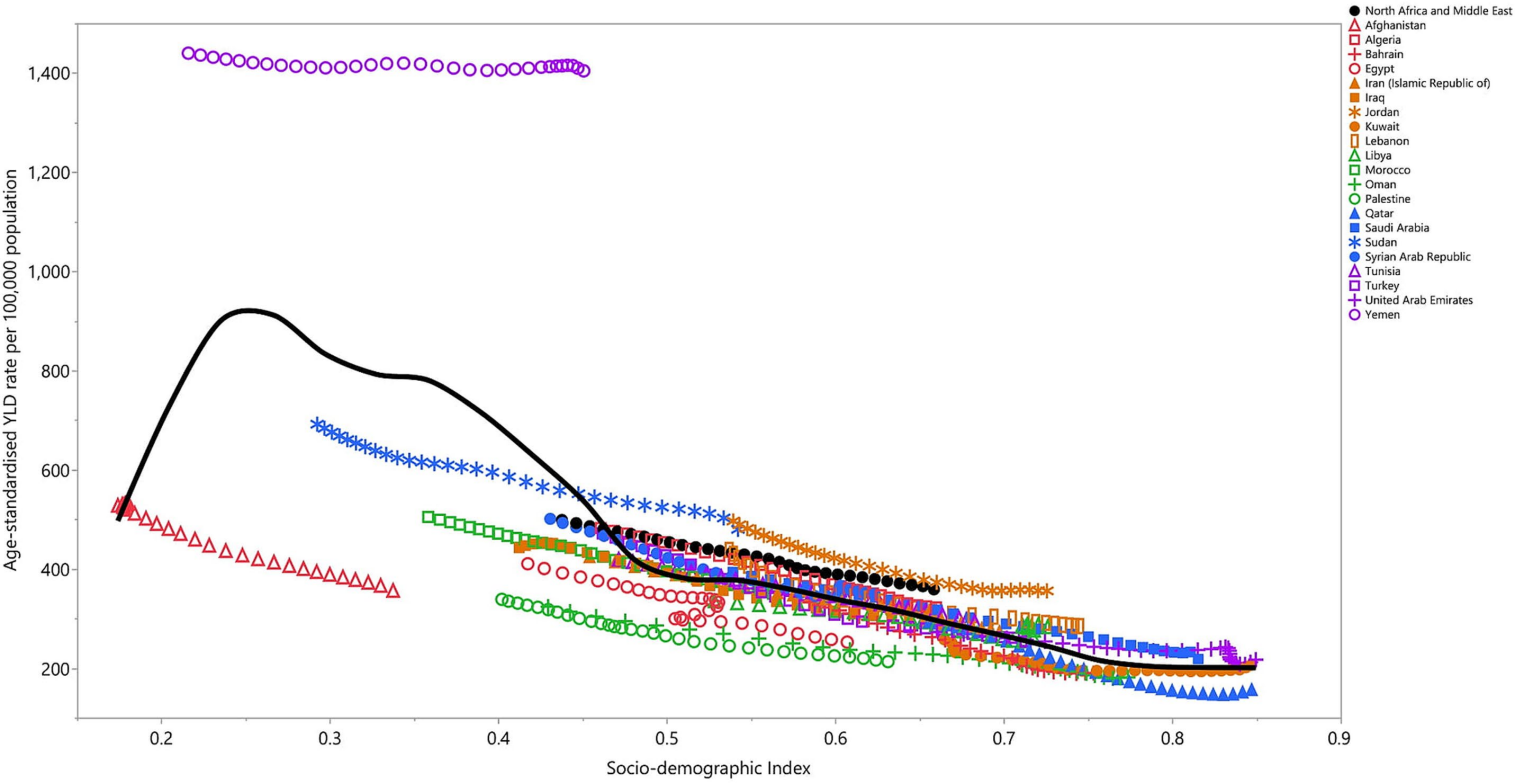
Age-standardised YLD rates of dietary iron deficiency for 21 countries and territories in the MENA region in 2021, plotted against the SDI. Expected values based on the SDI and disease rates in all locations are represented by the black line. Each point reflects the observed age-standardised DALY rate for each country. YLD, years lived with disability (generated from data available from http://ghdx.healthdata.org/gbd-results-tool).

The correlation between the SDI and age-standardised YLD rate in the MENA region from 1990 to 2021 exhibited a non-linear pattern. The burden of iron deficiency increased with rising socio-economic development up to an SDI of approximately 0.25, after which it gradually declined with higher SDI levels. Up to an SDI of around 0.46, the regional rate was below the predicted level; at higher SDI levels, it exceeded the expected level. Furthermore, countries such as Jordan, Algeria, Saudi Arabia, Lebanon, and the United Arab Emirates experienced iron deficiency burden above the regional average. In contrast, Afghanistan, Palestine, Egypt, Kuwait, and Oman had ID burdens below the regional average (Figure 5).

## 4 Discussion

The burden of dietary iron deficiency in the MENA region, as evidenced by the GBD data for 1990–2021, highlights persistent challenges in public health. The results reveal that despite some improvements in socio-economic conditions, the prevalence and YLD due to ID remain alarmingly high across many countries in the region, particularly in low-income countries. In this discussion, we will explore the epidemiological trends of iron deficiency, compare them to global patterns, discuss the socio-demographic factors influencing iron deficiency, and explore age and sex differences. Additionally, we will evaluate public health measures, while considering the limitations and potential solutions for mitigating iron deficiency in the MENA region.

### 4.1 Epidemiological trends

The global burden of iron deficiency has shown modest declines between 1990 and 2021, particularly in high-SDI countries. The global GBD 2021 study also identified dietary iron deficiency as the primary cause of anaemia in the MENA region (2). Unlike these global declines, the MENA region, particularly low-SDI countries, has not experienced comparable reductions, a pattern consistent with findings from other low- and middle-income regions, including South Africa (21). This slow progress in the MENA region reflects broader global challenges in addressing iron deficiency, particularly in regions affected by political instability, conflict, and limited healthcare access. In middle- and high-SDI countries in the MENA region, rising urbanisation and affluence, as well as climate changes, have led to increased consumption of nutrient-poor, processed foods and higher rates of obesity, which exacerbate the prevalence of iron deficiency (22–25).

In 2021, the MENA region recorded 88.9 million cases of iron deficiency, with an age-standardised prevalence of 14368.2 per 100,000 individuals, indicating no significant change since 1990. The prevalence of iron deficiency in the region represents a substantial public health burden. Despite slight regional variations, countries like Egypt, Yemen, and Turkey exhibited the highest number of cases, with Yemen and Sudan showing the highest rates. On the other hand, countries like Qatar, Bahrain, and Kuwait had relatively low prevalence. The higher burden in countries such as Yemen and Sudan, which also face conflict and socio-economic challenges, suggests a strong link between poverty, food insecurity, and iron deficiency prevalence. These findings are in line with previous studies indicating that populations with lower socio-economic status tend to have poorer access to iron-rich foods and healthcare (7, 10, 26).

The 26% decline in iron deficiency prevalence since 1990 reflects some improvement in nutritional interventions and socio-economic development in parts of the MENA region. However, the persistence of iron deficiency at such high levels points to the need for sustained and targeted public health efforts. The GBD data also showed a 28% decline in YLDs, with the current age-standardised YLD rate of 359.7 per 100,000. These reductions may be attributed to improved healthcare systems, fortification programmes, and increased awareness of micronutrient deficiencies. However, the continuing burden of iron deficiency underscores the complexity of addressing micronutrient deficiencies in a region characterised by significant disparities in economic development, health infrastructure, and access to education and healthcare (6, 26).

### 4.2 Country-level disparities

The country-level analysis highlights significant disparities in iron deficiency prevalence within the MENA region. Yemen, Egypt, and Sudan, which are among the region’s poorest countries, had the highest iron deficiency rates. These countries face chronic challenges related to food security, conflict, and limited healthcare access (4, 27, 28). For instance, Yemen’s prevalence of 30146.5 per 100,000 is significantly higher than that of countries like Qatar and Bahrain, which have lower prevalence due to better socio-economic conditions and stronger healthcare systems. The disparities between countries with high and low age-standardised rates reflect the influence of socio-economic factors on health outcomes. This is consistent with studies showing that iron deficiency disproportionately affects populations with limited access to healthcare and nutritious foods, especially in regions experiencing political instability and economic hardship (11, 29).

Countries with lower prevalence and YLD rates, such as Qatar, Kuwait, and Saudi Arabia, benefit from higher per capita income and stronger healthcare infrastructures, allowing for more effective prevention and management of iron deficiency. These countries have invested in food fortification programmes, public health campaigns, and maternal and child health services, which have contributed to lower iron deficiency burden. However, even in wealthier nations, certain population groups, such as women of reproductive age, remain vulnerable to iron deficiency, particularly during pregnancy, highlighting the need for ongoing public health efforts to address micronutrient deficiencies (11).

### 4.3 Gender disparities

The results also revealed a significant sex disparity in the burden of dietary iron deficiency across the MENA region, with females generally exhibiting higher prevalence and YLDs rates compared to males. This finding aligns with global trends that consistently highlight a higher prevalence of iron deficiency among women, particularly in reproductive age groups (26). Biological factors, such as menstrual blood loss, pregnancy, and lactation, contribute to this disparity (10, 19, 30).

According to GBD 2021 global results, women worldwide experience higher rates of anaemia and iron deficiency due to physiological factors like menstruation and pregnancy. The slower reduction in iron deficiency among women in low-SDI MENA countries, such as Yemen and Afghanistan, reflects global trends (2). Similarly, Turawa et al. (21) found that in South Africa, the prevalence of iron deficiency and anaemia in women of reproductive age has remained consistently high over the past two decades. In both global and regional contexts, these findings underscore the need for targeted interventions to address gender-based disparities in nutritional health. Additionally, gender-specific socio-cultural factors in the MENA region—such as dietary restrictions and limited access to healthcare for women in some areas—may exacerbate the risk of iron deficiency among females (29).

Importantly, the correlation between reproductive health and iron deficiency cannot be ignored, especially in the context of anaemia in pregnant women. Studies indicate that iron deficiency during pregnancy can have severe consequences for both mother and child, including increased risk of maternal mortality, preterm birth, and low birth weight (31, 32). Given the high fertility rates in some MENA countries and ongoing humanitarian crises in places like Yemen and Sudan face, the delivery of maternal healthcare and iron supplementation programmes is further hampered by unstable infrastructures (6, 33, 34). Current interventions aimed at improving iron intake, such as iron supplementation during pregnancy and lactation, remain critical but may need scaling and adaptation to be more accessible, especially in low-SDI countries like Yemen and Sudan.

It is also notable that men in the age groups 20–34 and 85–89 years exhibited YLD rates above the global average, with men aged 25–29 showing the highest MENA-to-global YLD ratio. These data indicate a need for a more nuanced exploration of the iron deficiency burden in men, who are often considered at lower risk than women. However, socioeconomic factors, lifestyle choices, and specific health conditions, such as gastrointestinal diseases or chronic infections, could predispose certain male age groups to ID (35).

### 4.4 Age-specific burden and its implications

The burden of iron deficiency among children under 5 years of age in the MENA region remains high, aligning with global trends. GBD 2021 global findings emphasise that children in low- and middle-income countries remain vulnerable to iron deficiency due to poor weaning practices and malnutrition (2, 36). Children are particularly vulnerable to iron deficiency due to rapid growth demands, and its consequences can be severe, impacting cognitive development, immune function, and overall growth (26, 37). The MENA region’s high prevalence of childhood iron deficiency can be attributed to poor dietary quality, insufficient iron intake from complementary feeding, and high rates of infections that impair nutrient absorption (38, 39). These findings indicate that the MENA region shares many of the same challenges in addressing early childhood iron deficiency as other regions, despite global efforts to reduce this burden. For children under five, the provision of iron-fortified foods, supplementation, and public health campaigns promoting better dietary practices could help reduce the future burden of ID (39, 40).

In contrast to global reductions observed in some higher-SDI regions, the burden of iron deficiency in elderly populations within the MENA region has been rising. Gardner et al. (2) suggest that global improvements in elderly nutrition are more pronounced in regions with better healthcare access and nutritional programmes. However, as observed in the MENA region and similar to trends reported in South Africa by Turawa et al. (21), iron deficiency among the elderly continues to be a growing concern.

Our results indicate that males in the age group 85–89 are at higher risk for disability due to iron deficiency, echoing findings from previous studies. Ageing populations experience a natural decline in nutritional absorption efficiency, and comorbidities such as chronic diseases can further exacerbate the risk of iron deficiency (41). As the region undergoes demographic transitions, with increasing life expectancy and a growing proportion of elderly citizens, it is imperative to implement targeted nutritional interventions to mitigate the health impact of iron deficiency in this demographic (12, 42).

### 4.5 Socio-demographic Index

The correlation between SDI and the burden of iron deficiency in the MENA region reveals a non-linear relationship, where the burden of iron deficiency increases with rising socio-economic development up to an SDI of 0.25, after which it gradually declines. This trend highlights the complex interplay between socio-economic factors and iron deficiency prevalence. The non-linear relationship between SDI and iron deficiency burden underscores the fact that, while economic development and improved living standards can reduce the prevalence of iron deficiency, certain populations in middle- to high-SDI countries may still experience a disproportionate burden of the condition. This phenomenon may be related to dietary transitions seen in more developed countries, where traditional nutrient-rich diets are often replaced by more processed, iron-poor foods (12).

In lower-SDI countries such as Yemen, Sudan, and Afghanistan, the high burden of iron deficiency is unsurprising, given the context of political instability, conflict, and food insecurity. These countries are characterised by limited access to healthcare services, poor maternal and child health outcomes, and high levels of poverty, all of which contribute to the prevalence of iron deficiency. Globally, GBD 2021 results highlight that regions with low SDI experience the greatest burden of iron deficiency and anaemia, particularly among women and children (2). For these countries, policy recommendations often center around humanitarian aid, food fortification programmes, and broader efforts to address poverty and inequities in access to healthcare (43). In contrast, as countries move towards a higher SDI, better healthcare access, food fortification programmes, and improved living conditions contribute to the decline in the iron deficiency burden (44, 45).

Meanwhile, countries with higher SDI levels, such as Jordan, Saudi Arabia, and the UAE, have shown a higher-than-expected iron deficiency burden, potentially pointing to other determinants such as lifestyle factors (e.g., high rates of obesity) and dietary habits (42). This pattern of rising iron deficiency in affluent populations, driven by processed and nutrient-poor diets, is noted on a global scale (2). These findings suggest that even in wealthier nations, nutrition policies must be attuned to changes in diet and lifestyle that may negate some of the benefits of higher socioeconomic development.

### 4.6 Public health implications and strategies

The high burden of ID in the MENA region necessitates comprehensive public health strategies to mitigate the impact of this condition (46). Firstly, the large variation in prevalence and YLD rates across countries underscores the need for country-specific interventions tailored to the unique socioeconomic, cultural, and health system contexts of each nation. In countries with a high burden, like Yemen and Sudan, where food insecurity and malnutrition are widespread, large-scale food fortification programmes and improvements in maternal and child health services could play a significant role in reducing the prevalence of iron deficiency (6, 11). Such programmes have been shown to be cost-effective in other low- and middle-income countries, providing a sustainable solution to micronutrient deficiencies (47). In contrast, countries like Saudi Arabia and the UAE may benefit from targeted public health campaigns aimed at promoting healthier dietary habits and addressing micronutrient deficiencies in otherwise affluent populations (48, 49).

Secondly, the strong association between reproductive health and iron deficiency in women highlights the importance of integrating iron deficiency prevention into broader maternal and child health initiatives. Public health campaigns focused on educating communities about the importance of iron-rich diets, particularly for women and children, are crucial. Addressing gender disparities in dietary habits, as observed in Tunisia and other MENA countries, could also contribute to reducing the ID burden among women (12). Furthermore, interventions such as providing iron supplements during antenatal care, fortifying staple foods with iron, and addressing underlying causes of iron deficiency in women, such as menstruation and pregnancy-related blood loss, are essential components of comprehensive health policies. Meeting the nutritional needs of young children, particularly through breastfeeding promotion, complementary feeding education, and iron fortification of weaning foods, as well as improving the diagnosis and management of iron deficiency through healthcare services, could mitigate the long-term impact of iron deficiency in the region.

Lastly, the correlation between SDI and iron deficiency burden suggests that economic development alone may not be sufficient to address micronutrient deficiencies, particularly in countries undergoing dietary and lifestyle transitions. Public health efforts should therefore be multi-sectoral, encompassing policies that promote food fortification, ensure the availability and affordability of iron-rich foods, and raise awareness about the importance of nutrition in preventing chronic diseases. Governments should also invest in robust healthcare systems capable of diagnosing and treating iron deficiency at early stages to prevent long-term disability.

### 4.7 Limitations and future directions

While the GBD data provides a comprehensive overview of the iron deficiency burden in the MENA region, there are limitations to consider. Firstly, the reliance on national-level data may obscure important subnational variations in iron deficiency prevalence and burden, particularly in countries with large rural populations or ongoing conflicts, such as Yemen and Sudan. Future studies should aim to disaggregate data by subnational regions and residential setting (rural or urban) to better understand geographic disparities in iron deficiency burden and identify high-risk areas for targeted interventions. Secondly, the GBD data does not capture the full complexity of factors contributing to iron deficiency, such as the role of genetic conditions, chronic diseases, and the impact of environmental factors like food availability and quality. Future research should explore these factors in greater detail to provide a more comprehensive understanding of the drivers of iron deficiency in the MENA region. Thirdly, the GBD estimates come with wide uncertainty intervals, especially in low- and middle-income countries, which impacts the precision of the findings. While these estimates offer important insights, the substantial uncertainty somewhat constrains their overall accuracy. Additionally, the modeling process relies on assumptions about age, cohort, and period effects, which can sometimes simplify or miss the impact of more intricate social and environmental influences. Consequently, future research could benefit from considering alternative models and methods that better capture these complexities. Finally, while public health interventions such as iron supplementation and food fortification have proven effective in reducing iron deficiency, more research is needed to evaluate their long-term sustainability and impact, particularly in low-resource settings. Collaborations between governments, non-governmental organisations, and international bodies like the World Health Organization will be crucial for scaling up these interventions and ensuring that they reach the populations most in need.

## 5 Conclusion

Our findings underscore the significant burden of dietary iron deficiency across the MENA region, with pronounced disparities by age, sex, and socioeconomic status. Addressing this public health issue requires a comprehensive and tailored approach that takes into account the diverse health needs of the population, the socioeconomic challenges faced by low-SDI countries, and the shifting dietary patterns in more affluent nations. Policy-makers should prioritise nutrition-sensitive interventions that enhance food security, improve maternal and child health, and promote healthier dietary habits, with an emphasis on long-term sustainability and equity.

## Author’s note

This study is based on publicly available data and reflects the authors’ views, not those of the Institute for Health Metrics and Evaluation.

## Data Availability

The original contributions presented in the study are included in the article/supplementary material, further inquiries can be directed to the corresponding authors.

## Footnotes

^1^ http://ghdx.healthdata.org/gbd-2019/data-input-sources
